# Computational and experimental data on electrostatic density and stacking tendency of asymmetric cyanine 5 dyes

**DOI:** 10.1016/j.dib.2018.11.132

**Published:** 2018-11-29

**Authors:** S.J. Spa, A.W. Hensbergen, S. van der Wal, J. Kuil, F.W.B. van Leeuwen

**Affiliations:** a Interventional Molecular Imaging Laboratory, Department of Radiology, Leiden University Medical Center, the Netherlands; b Laboratory of BioNanoTechnology, Wageningen, the Netherlands; c Department of Clinical Pharmacy and Toxicology, Leiden University Medical Center, the Netherlands

**Keywords:** Fluorescence, Cyanine, Stacking, Electrostatic density

## Abstract

Far-red dyes such as cyanine 5 (Cy5) are gaining interest in (bio)medical diagnostics as they have promising features in terms of stability and brightness. Here, the electrostatic density and stacking tendency in different solvents of nine systematically altered asymmetrical Cy5 dyes are reported. In addition to this, the influence of molecular alterations on the vibronic coupling was reported. The data presented supplement to the recent study “The influence of systematic structure alterations on the photophysical properties and conjugation characteristics of asymmetric cyanine 5 dyes” (Spa et al., 2018).

**Specifications table**

**Table:** 

Subject area	*Chemistry*
More specific subject area	*Photochemistry*
Type of data	*Figures and graphs*
How data were acquired	Absorbance of cyanine 5 (Cy5) analogues was measured with the Ultrospec 3000 UV–Visible spectrophotometer (Pharmacia Biotech). Electrostatic densities were calculated using Wavefunction Spartan software
Data format	*Analyzed, computational*
Experimental factors	*4 mM stock solutions of the dyes were prepared in DMSO-d*_*6*_*containing 4 mM ethylene carbonate. Absorbance of the dyes was measured at variable concentrations and the obtained graphs normalized on concentration to acquire insight on the stacking behavior of various Cy5 dyes*
Experimental features	*The relationship between molecular design and stacking interactions based on the electrostatic interactions of Cy5 fluorophores was determined*
Data source location	*Leiden University Medical Center, Leiden, The Netherlands-*
Data accessibility	*The data are available within this article*
Related research article	Spa SJ, Hensbergen A.W., van der Wal S, Kuil J, van Leeuwen FWB. The influence of systematic structure alterations on the photophysical properties and conjugation characteristics of asymmetric cyanine 5 dyes. Dyes and Pigments.2018152:19–28 [1]

**Value of the data**

• The data give more insight into the stacking behavior and vibronic coupling of asymmetrical Cy5 dyes which can be used to optimize a tracer׳s molecular structure.
• Structural variations in asymmetrical Cy5 dyes influence the electron densities, which could help to understand the alterations in the photophysical and stacking characteristics of the dyes.

## 1 Data

**Fig. 1:**
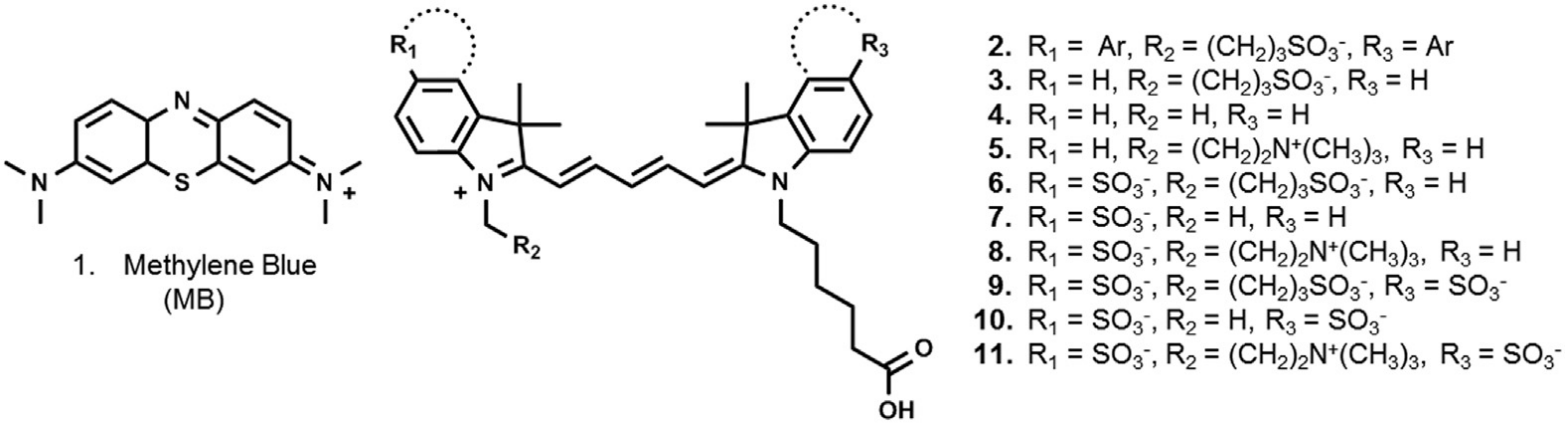
Structure of compounds **1**–**11** showing the structural variations and comparison to methylene blue (MB). R_1_ and R_3_ depict substituents on the aromatic ring on the indole, R_2_ depicts a substituent on the N-indole.

**Fig. 2:**
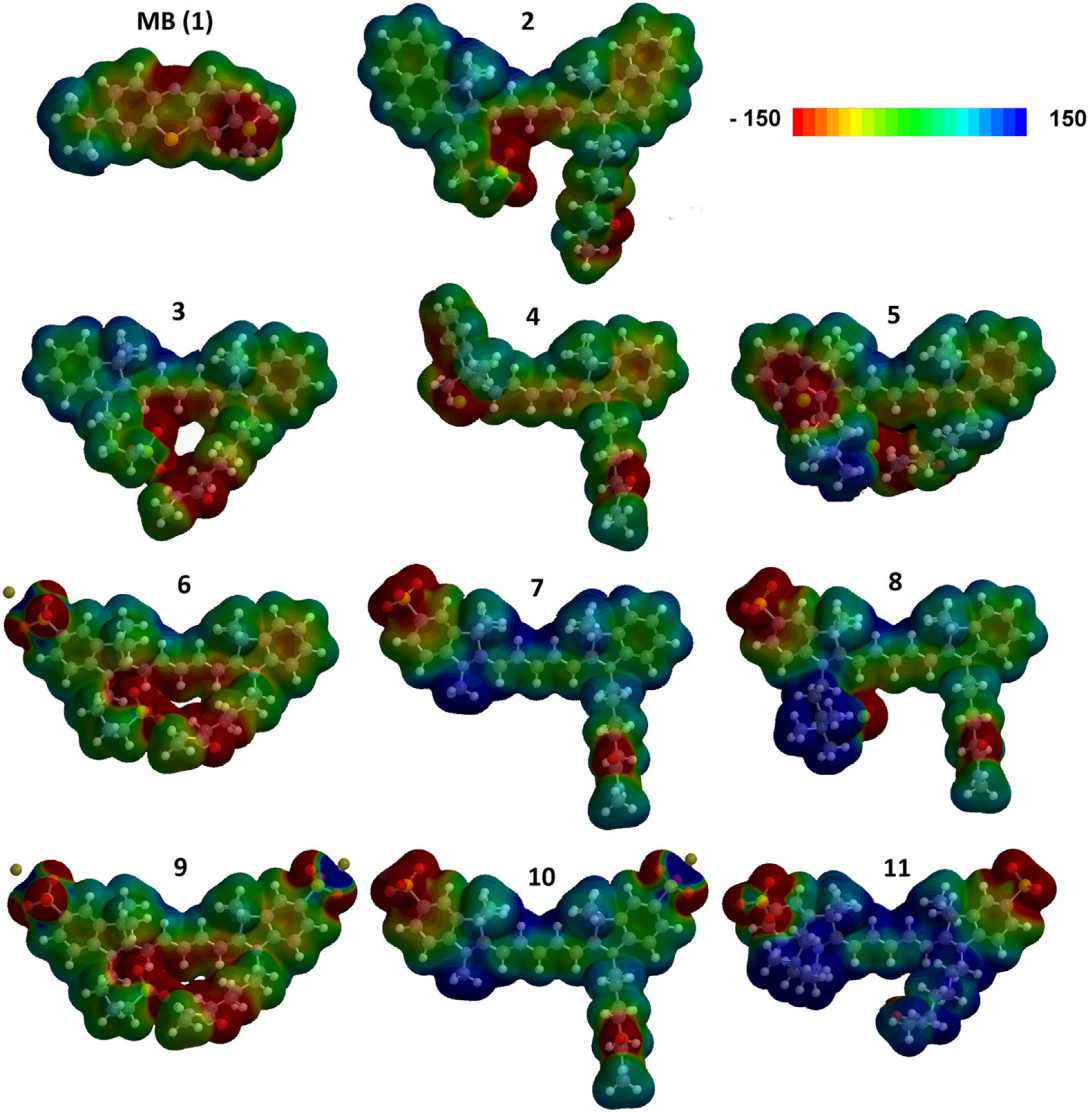
Electrostatic densities of compound **1**–**11**, overlaid over ball-and-stick models representing the theoretically most favored conformation (lowest electron potential). The electron densities on the molecules are visualized from red to blue, with red representing the electron-rich regions and blue the electron-poor regions (arbitrary units).

**Fig. 3:**
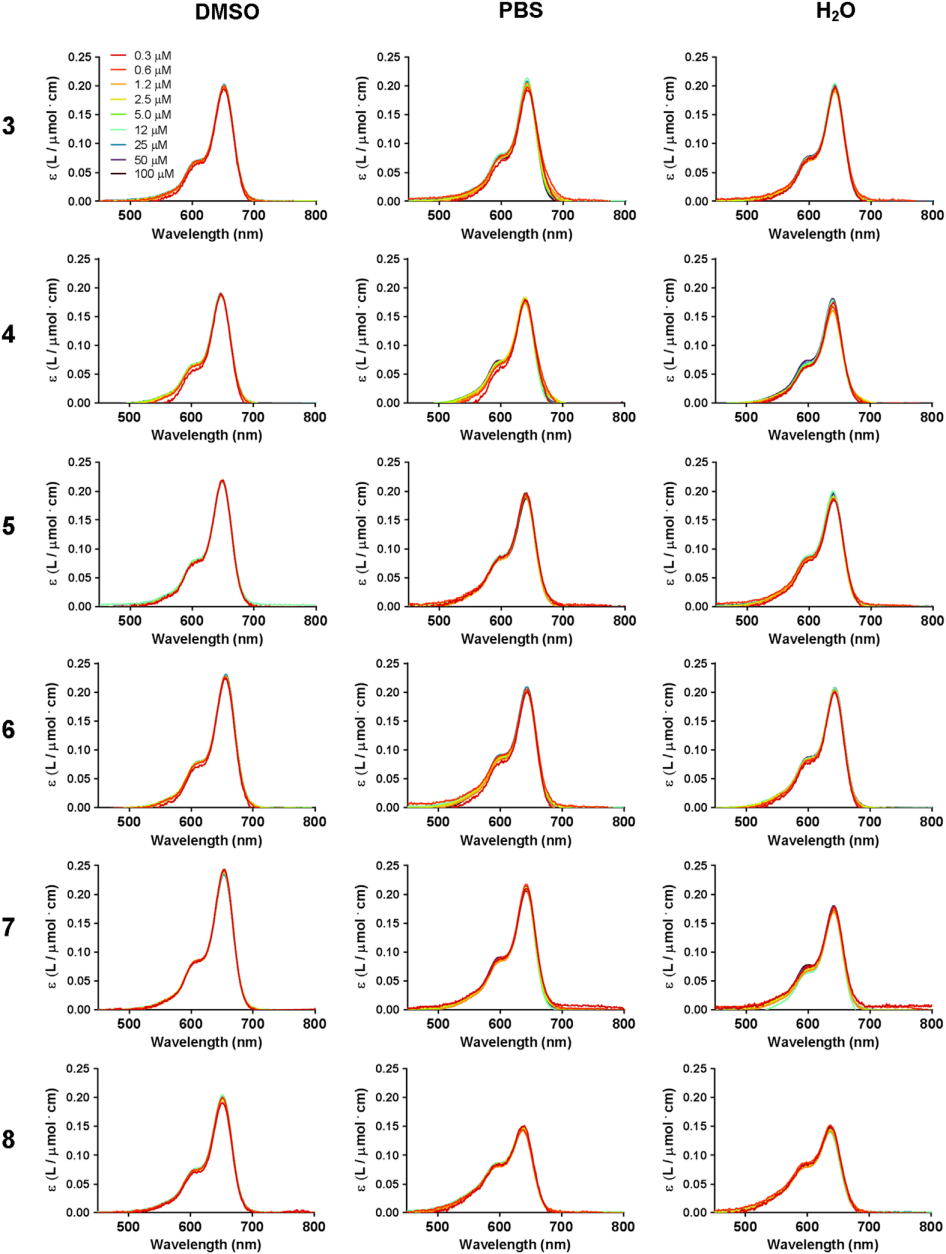
Stacking behavior of compounds **3**–**11** in DMSO, PBS and H_2_O measured at a concentration range of 0.3–100.0 µM (red to deep purple; concentration range and color coding is depicted in the top left graph).

The dataset of this data article provides information on the electron densities and stacking interactions of various cyanine 5 dyes. The Fig. 1, Fig. 2, Fig. 3, show the different molecular structures, electron densities and stacking behavior, respectively.

The evaluated fluorophores are depicted in Fig. 1. These structural analogues were analyzed to determine whether molecular substituents on the fluorophore influences their utility as an imaging label [1].

### 1.1 Electrostatic density and conformation of the compounds

Spartan modeling software (See 2.3) was used to calculate the most stable conformation, *i.e.*, lowest potential energy, and electron distribution for all synthesized dyes. Calculations were performed on the dyes including their counterions Cl^−^ or K^+^ to allow for comparison at neutral overall charges. Although the K^+^ ion is not included in the electron density cloud calculated by Spartan (Fig. 2), its electrostatic effect on the sulfonate was included.

### 1.2 Stacking of compound **2–11** in different solvents

The stacking tendency of compound **2**–**11** in DMSO, PBS and H_2_O was evaluated by measuring the dyes’ absorbance at various concentrations. Previous reports describe the stacking of methylene blue (**1**) [1] and this dye is therefore not included in this Data in Brief. The absorption spectra were assessed at concentrations ranging from 0.3 to 100.0 µM as the stacking characteristic of a dye is concentration-dependent. A hypsochromic or bathochromic shift is to be expected when dyes aggregate by H- or J-stacking, respectively [2].

Other than previous reports using symmetrical Cy7-analogues [3], the differences in the asymmetrical Cy5 analogues (Figs. 1 and 2) revealed no or only a minor stacking tendency for compounds **2**–**11**. It is interesting to note that for compounds **2**–**11** the height of the shoulder peak is found constant around 0.07 arbitrary units (AU) in DMSO and H_2_O, and around 0.08 AU in PBS (Fig. 3). The characteristic shoulder peak originates from vibronic coupling, *i.e.*, intramolecular electronic transitions [4]. Although the absorption efficiency, *i.e.*, molar extinction coefficient, varies by structurally altering the dye׳s molecular structure, the persistent height of the shoulder peak indicates that vibronic coupling was not affected by these alterations.

## 2 Experimental design, materials and methods

### 2.1 Materials and reagents

Synthesis of compound **2**–**11** was performed according to previous reports [1]. Absorbance spectra were measured with the Ultrospec 3000 UV–Visible spectrophotometer (Pharmacia Biotech) according to previously described methods [1].

### 2.2 Fluorophore stock solution

Stock solutions of the dyes (4 mM) were prepared in DMSO-d_6_ containing ethylene carbonate (4 mM; internal standard for NMR) and were stored at 4°C before further use as previously described [1].

### 2.3 Electron density modeling

Electrostatic potential mapping of the dyes was performed using Spartan ’16 (Wavefunction, Irvine USA) using the semi-empirical model and the PM6 method in the gas phase on the *N*-methylamide form of the fluorophores.

### 2.4 Stacking behavior of compound **1–11** in different solvents

The DMSO-d_6_ stock solutions of the dyes (see 2.2) were diluted to 100 µM in either DMSO, H_2_O or PBS. Subsequently, dilutions (50 µM and 5 µM) were made from these 100 µM solutions. Further dilution in the same solvent allowed for a final concentration range of 100.0, 50.0, 25.0, 12.5, 5.0, 2.5, 1.3, 0.6, and 0.3 µM, respectively. To keep the signal below 1.5 AU, absorption spectra of different concentrations were measured using different cuvettes: for concentrations ≤5.0 µM 1 mL disposable plastic cuvettes (*l* = 1 cm; Brand, Germany) were used, for concentrations ≥12.5 and ≤50.0 µM, quartz cuvettes (*l* = 0.1 cm; Hellma standard cell, Macro) were used, and for 100.0 µM concentrations two glass microscopy slides separated by a PET plastic spacer (*d* = 0.14 mm) were used. Spectra were measured at *t* = 10 min after preparation and normalized for cuvette path length and concentration.

## Funding source

The research was supported by the European Research Council (ERC) under the European Union׳s Seventh Framework Programme FP7/2007–2013 (Grant no. 2012-306890), and Netherlands Organization for Scientific Research STW-VIDI grant (Grant no. STW BGT11272).

## References

[1] Spa S.J., Hensbergen A., van der Wal S., Kuil J., van Leeuwen F.W.B. (2018). The influence of systematic structure alterations on the photophysical properties and conjugation characteristics of asymmetric cyanine 5 dyes. Dyes Pigment..

[2] Mishra B.K. (2007). Cyanine dyes: self aggregation and behaviour in surfactants a review. J. Surf. Sci. Technol..

[3] Van Der Wal S., Kuil J., Valentijn A.R.P.M., Van Leeuwen F.W.B. (2016). Synthesis and systematic evaluation of symmetric sulfonated centrally C–C bonded cyanine near-infrared dyes for protein labelling. Dyes Pigment..

[4] Zarow A., Shin Y. (2009). Cyanine dyes: fine structures in their absorption spectra. Am. J. Undergrad. Res..

